# Supplementary material for: End Sequence Analysis Toolkit (ESAT) expands the extractable information from single-cell RNA-seq data
Source: Genome Res. 2016 Oct;26(10):1397–410. doi: 10.1101/gr.207902.116 (PMC5052061; doi:10.1101/gr.207902.116)
Supplement: Supplemental Material [file supp_gr.207902.116_Supp_Materials.tar.gz › ESAT-esat_v0.1/doc/html/classbroad_1_1pda_1_1samtools_1_1_filter_fastq_reads.html]

ESAT: broad.pda.samtools.FilterFastqReads Class Reference


ESAT


- Main Page
- Related Pages
- Packages
- Classes
- Files

- Class List
- Class Index
- Class Hierarchy
- Class Members

All Classes Namespaces Files Functions Variables Enumerator Pages

- broad
- pda
- samtools
- FilterFastqReads

Public Member Functions |
Static Public Member Functions |
Public Attributes |
Protected Member Functions |
List of all members

broad.pda.samtools.FilterFastqReads Class Reference

Inheritance diagram for broad.pda.samtools.FilterFastqReads:

[legend]

Collaboration diagram for broad.pda.samtools.FilterFastqReads:

[legend]

|  |  |
| --- | --- |
| Public Member Functions | |
| String | getReadName (final String fastaqHeader) |
|  | |

|  |  |
| --- | --- |
| Static Public Member Functions | |
| static void | main (final String[] args) |
|  | |

|  |  |
| --- | --- |
| Public Attributes | |
| String | USAGE = "Filters FASTQ files to remove reads matching the provided read name list." |
|  | |
| File | INPUT\_1 |
|  | |
| File | INPUT\_2 = null |
|  | |
| File | OUTPUT\_1 |
|  | |
| File | OUTPUT\_2 = null |
|  | |
| File | READ\_LIST\_FILE |
|  | |

|  |  |
| --- | --- |
| Protected Member Functions | |
| int | doWork () |
|  | |

## Member Function Documentation

|  |  |  |  |  |
| --- | --- | --- | --- | --- |
| int broad.pda.samtools.FilterFastqReads.doWork | ( |  | ) |  |

protected

Here is the call graph for this function:

|  |  |  |  |  |  |
| --- | --- | --- | --- | --- | --- |
| String broad.pda.samtools.FilterFastqReads.getReadName | ( | final String | *fastaqHeader* | ) |  |

Here is the caller graph for this function:

|  |  |  |  |  |  |
| --- | --- | --- | --- | --- | --- |
| static void broad.pda.samtools.FilterFastqReads.main | ( | final String[] | *args* | ) |  |

static

Stock main method.

Parameters
:   |  |  |
    | --- | --- |
    | args | main arguments |

## Member Data Documentation

File broad.pda.samtools.FilterFastqReads.INPUT\_1

File broad.pda.samtools.FilterFastqReads.INPUT\_2 = null

File broad.pda.samtools.FilterFastqReads.OUTPUT\_1

File broad.pda.samtools.FilterFastqReads.OUTPUT\_2 = null

File broad.pda.samtools.FilterFastqReads.READ\_LIST\_FILE

String broad.pda.samtools.FilterFastqReads.USAGE = "Filters FASTQ files to remove reads matching the provided read name list."

---

The documentation for this class was generated from the following file:

- java/broad/pda/samtools/FilterFastqReads.java


---

Generated on Fri Aug 15 2014 15:11:01 for ESAT by  

 1.8.7
